# Remote physiological monitoring provides unique insights on the cardiovascular performance and stress responses of freely swimming rainbow trout in aquaculture

**DOI:** 10.1038/s41598-019-45657-3

**Published:** 2019-06-24

**Authors:** Jeroen Brijs, Erik Sandblom, Michael Axelsson, Kristina Sundell, Henrik Sundh, Anders Kiessling, Charlotte Berg, Albin Gräns

**Affiliations:** 1 Department of Animal Environment and Health, Swedish University of Agricultural Sciences, Skara, SE-532 31 Sweden; 2 Department of Biological and Environmental Sciences, University of Gothenburg, Gothenburg, SE-405-30 Sweden; 3 Department of Animal Nutrition and Management, Swedish University of Agricultural Sciences, Uppsala, SE-750 07 Sweden

**Keywords:** Cardiovascular biology, Biological techniques, Animal physiology, Animal behaviour, Circulation

## Abstract

Investigating the mechanisms that fish employ to maintain homeostasis in their everyday life requires measurements of physiological and behavioural responses in the field. With multivariate bio-loggers, we continuously measured gastrointestinal blood flow (GBF), heart rate, activity and body temperature in rainbow trout (*Oncorhynchus mykiss*) swimming freely amongst ~5000 conspecifics in a sea cage. Our findings clearly demonstrate that while both acute aquaculture-related stress and spontaneous activity resulted in transient reductions in GBF (*i*.*e*. reductions of up to 65%), recovery from stressful handling practices subsequently involved a substantial and prolonged gastrointestinal hyperemia far beyond the level observed prior to the stressor. The gastrointestinal hyperemia may be necessary to repair the damage to the gastrointestinal tract caused by acute stress. Furthermore, heart rate responses to acute stress or voluntary activity differed depending on the individual’s physiological state. Stressed fish (*i*.*e*. mean heart rates >70 beats min^−1^) exhibited a bradycardic response to acute stress or activity, whereas fish with mean heart rates <60 beats min^−1^ instead demonstrated strong tachycardic responses. Remote monitoring of physiological and behavioural variables using bio-loggers can provide unique insights into ‘real-life’ responses of animals, which can largely differ from the responses observed in confined laboratory settings.

## Introduction

To discover the mechanisms that free-living organisms employ to maintain homeostasis in their everyday life, physiological responses need to be measured in the field rather than in controlled laboratory environments^1,2^. However, this is challenging, as most physiological recording techniques require the animal to be physically connected to the recording equipment^1,2^. Fortunately, rapid development and miniaturization of bio-loggers and bio-telemetry systems presents a solution, as it allows the remote recording of physiological data in free-living organisms over long uninterrupted periods^1–7^. In addition to a range of invertebrates, amphibians, reptiles, birds and mammals; bio-logging and bio-telemetric techniques have also successfully been applied on a range of fish species in the wild and in captivity^4–7^. In fish, bio-loggers are particularly valuable as they allow high-resolution recordings of unattenuated signals when compared to radio-based telemetry devices (as radio signal transmission is severely attenuated in water)^4–7^. In contrast to radio signals, sound waves propagate more efficiently in water than in air and thus acoustic-based telemetry also represents a useful tool for recording physiological responses in freely swimming fish^4–7^. An advantage of acoustic-based telemetry is that data does not need to be physically retrieved since it is continuously transmitted in ‘real-time’ throughout the study. However, due to current technological limitations, the resolution of data that can be transmitted via acoustic-based telemetric devices is substantially lower than that collected using bio-loggers^4–7^.

A specific research area that would benefit from the application of bio-logging and bio-telemetric devices is the study of stress physiology. The use of these devices for evaluating the effects of stress on key physiological and behavioural variables of freely swimming fish is the way forward, since fish have the freedom to move around and exhibit a full repertoire of natural behaviours in response to environmental and/or anthropogenic stressors^1,2^. Furthermore, confounding laboratory artefacts such as the physical connection to recording equipment, visual and auditory laboratory disturbances, and confinement stress are eliminated^5–7^. Stress can be defined as factors that threatens an animal’s state of homeostasis, which induces behavioural and physiological responses to re-establish homeostasis (*i*.*e*. allostasis^8,9^). Typically, in response to an acute stressor, the hypothalamic-pituitary-interrenal axis and autonomic nervous system are activated to release catecholamines and corticosteroids^10,11^. This in turn elicits a range of secondary responses such as heightened cardiorespiratory activity, redistribution of blood flow to oxygen demanding tissues, splenic release of red blood cells and mobilization of energy stores, which all serve to increase an individual’s chance of survival in threatening situations^9,12^. However, detrimental tertiary stress responses such as impaired appetite, growth, swimming ability, immune function, behavioural repertoire and reproductive ability may occur when fish are chronically stressed and continuously subjected to high allostatic loads^9,12,13^. When fish display such tertiary stress responses in aquaculture, it is often considered as signs of compromised fish welfare, which has become of increasing concern for consumers, producers, interest groups and authorities due to ethical and/or economic reasons^12,14,15^. Finding alternative welfare indicators that do not rely on visual observations of fish or on the occurrence of detrimental tertiary stress effects are therefore particularly important in the aquaculture industry^12,13^. Thus, there is substantial interest in developing techniques that allow the continuous measurement of physiological indicators in fish, as this would allow the determination of the origin/cause of the stress response or for an intervention to take place before stress becomes distress and turns detrimental^9,13,16^.

The remote measurement of heart rate, a central cardiovascular variable affected by most behavioural responses and environmental perturbations, has been widely used to determine and quantify acute stress responses of fish subjected to various aquaculture and commercial/recreational fisheries practices in the field^17–21^. However, a few laboratory studies have demonstrated a strong negative correlation between routine heart rates and the scope to increase heart rate in response to enforced activity, which suggests that heart rate responses of fish can differ depending on the physiological state of the animal^18,22–24^. Chronically stressed fish may therefore not respond to environmental and/or anthropogenic challenges in the same manner as unstressed fish would, and so further understanding of the environmental factors and integrative physiological principles that affect heart rate of freely swimming fish is necessary^1,2,6^.

Another early physiological indicator of stress, and potential method for assessing the welfare of farmed fish, are changes in gastrointestinal blood flow (GBF)^4,25^. GBF is known to decrease drastically in fish in response to acute stress, as blood flow is prioritized to other oxygen demanding organs and muscle tissues to support the fight-or-flight response^26–28^. GBF is essential for sustaining the oxygenation and nutritional levels of the gastrointestinal tract, a multifunctional and highly metabolically active organ system responsible for a wide range of essential homeostatic processes^28–30^. In addition, GBF is also responsible for transporting metabolic waste products and assimilated nutrients, water and ions from the gastrointestinal tract to other sites for excretion or utilisation^30^. Thus, both acute and chronic environmental and/or anthropogenic stressors that alter GBF have the potential to significantly impact fish performance, health and energy expenditure^4,28–31^. A relatively recent innovation is the development of a fully implantable, multivariate bio-logger system (*i*.*e*. a bio-telemetric implant combined with a logger) that has the capacity to simultaneously measure blood flow (*i*.*e*. GBF), electrocardiogram (ECG), activity (via acceleration), and body temperature in freely swimming fish (Transonic EndoGear3 Implant and custom-built Logger, Transonic Systems Inc, Ithaca, New York, USA). With this novel system, it is now possible to continuously monitor multiple physiological processes in fish over extended periods of time in semi-natural and aquaculture settings. This will provide unique insights into the integrated physiological and behavioural responses of freely swimming fish interacting with each other and their environment over time^1,6,7^.

By exploiting this technology, the overall aim of the present study was to investigate physiological and behavioural processes of unrestrained fish in a commercial aquaculture sea cage and their responses to various handling stressors and environmental variables. In addition, we aimed to investigate whether *in vivo* monitoring of GBF in fish could be used as an early indicator of stress, as well as further investigating the suitability of *in vivo* monitoring of heart rate as a tool for assessing the welfare of rainbow trout (*Oncorhynchus mykiss* Walbaum 1972) in aquaculture. Specifically, by using surgically implanted bio-loggers in focal fish freely swimming among un-instrumented conspecifics in a commercial aquaculture sea cage, we could simultaneously monitor GBF, ECG, activity and body temperature in order to assess the severity of stress responses during a range of common farming practices (*e*.*g*. crowding, brailing and well-boat transport), as well as the physiological dynamics of recovery following the stressors. This information is invaluable for answering questions concerning fundamental stress biology in fish, as well as for the development of specific recommendations for future legislation aiming to improve fish welfare in aquaculture.

## Results

### Cardiovascular responses of freely swimming trout during a series of common aquaculture practices associated with transporting fish from slaughterhouse to the sea cage

**Figure 1:**
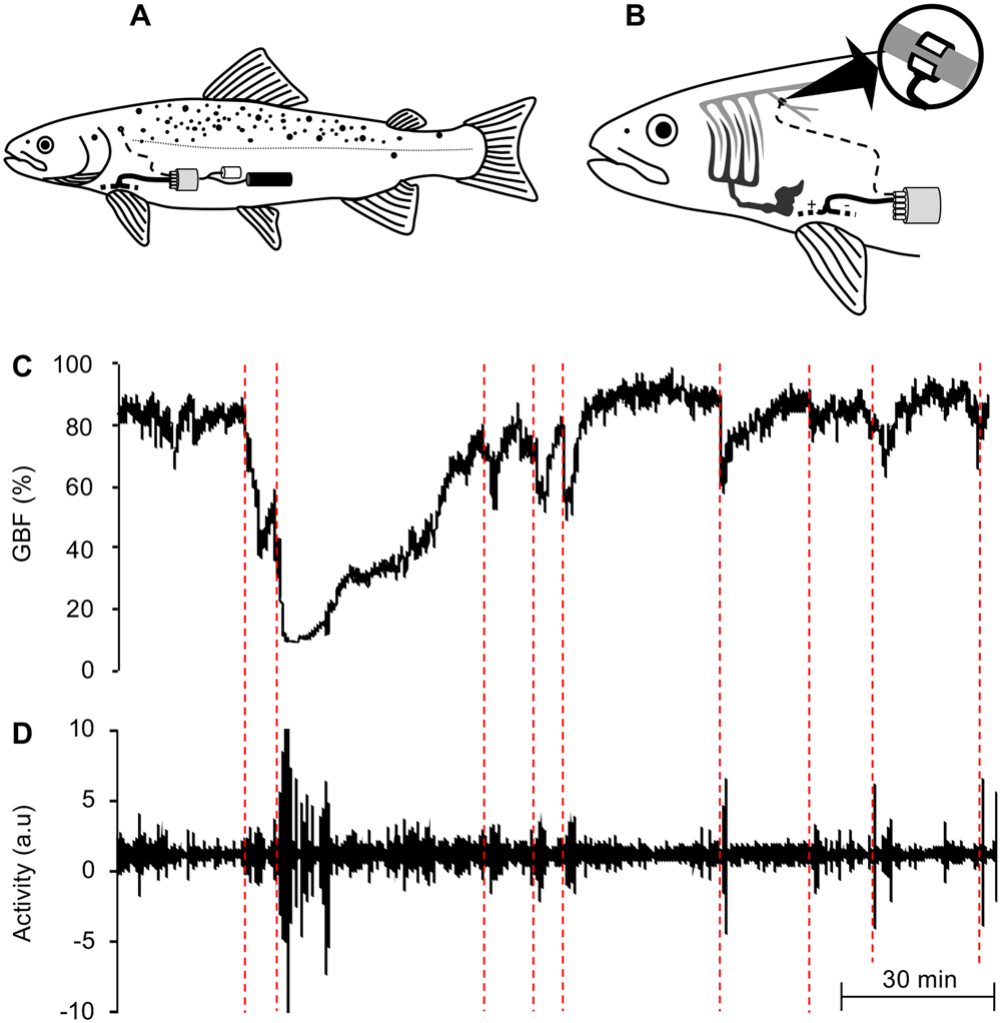
Positioning of the bio-logger within the rainbow trout (*Oncorhynchus mykiss*) and examples of the high-resolution recordings that can be obtained from the device. (**A**) Schematic showing the position of the implant (grey cylinder), battery pack (black cylinder) and data logger (white cylinder) in the abdominal cavity of a rainbow trout. (**B**) Schematic demonstrating the position of the blood flow probe on the coeliacomesenteric artery (enlarged), as well as the position of the two ECG-electrodes between the pectoral fins. Representative trace of (**C**) gastrointestinal blood flow (GBF) and (**D**) activity simultaneously recorded in a rainbow trout over a 5.5 h period, which clearly demonstrates the reductions that occur in GBF in response to activity (marked with the red dashed lines). Schematics (**A**,**B**) were drawn by Albin Gräns.

Signals from the bio-loggers located within the trout (Fig. 1A,B) were strong and clear, and showed no deterioration during bursts of activity. The high-resolution measurements obtained by the bio-loggers clearly demonstrate the simultaneous reductions that occur in GBF during bursts of activity (Fig. 1C,D). Visual inspection of GBF and activity traces revealed that the magnitude of the reductions in GBF were generally associated with the intensity and duration of the bursts of activity (Fig. 1C,D).

**Figure 2:**
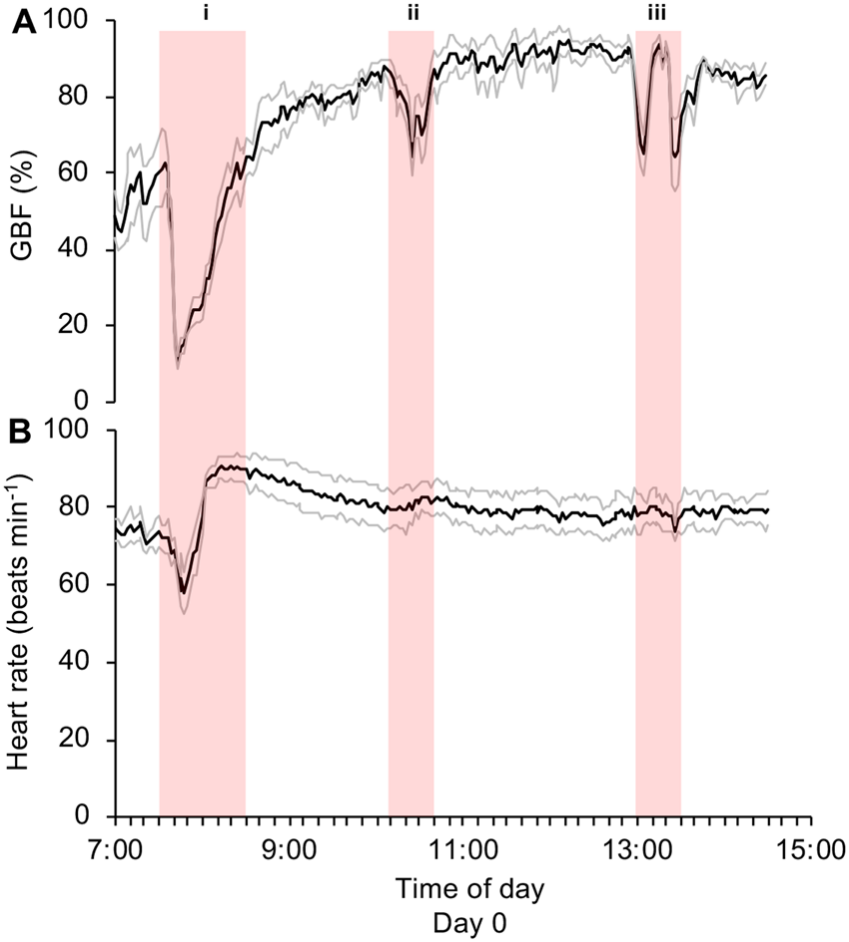
Cardiovascular responses of freely swimming rainbow trout during a series of common aquaculture practices following the surgical implantation of bio-loggers. (**A**) Mean gastrointestinal blood flow (GBF) and (**B**) heart rate of freely swimming rainbow trout subjected to common aquaculture practices including (i) transportation, (ii) brailing from the holding cage to the sea cage, and (iii) a scheduled feeding event for the ~5000 conspecifics inhabiting the sea cage. Means were calculated every 2 minutes during the 7.5 h period (n = 4, black line = mean, grey lines = ±s.e.m.).

There were clearly visible changes in both GBF and heart rate of trout (n = 4) in response to common aquaculture practices on day 0 (*i*.*e*. transportation, brailing and a scheduled feeding event; see i-iii in Fig. 2). The initiation of the transportation process (*i*.*e*. presence of humans around holding cage, boat noise and towing of cage) resulted in significant reductions in GBF (from ~63% to 10%; Fig. 2A) and heart rate (decrease of ~12 beats min^−1^; Fig. 2B, Table 1). Following these initial reductions, GBF and heart rate gradually increased during transportation to reach levels that were not significantly different from those observed prior to the stressor (Fig. 2, Table 1).

**Table 1:** Cardiovascular responses of rainbow trout during a series of common aquaculture practices following the surgical implantation of bio-loggers.

Stressor	GBF (%)	Heart rate (beats min^−1^)
**Transportation**	**(F**_**2,6**_ ** = 38.499, p < 0.001)**	**(F**_**2,6**_ ** = 20.790, p = 0.002)**
Directly before transportation	63 ± 8^a^	70 ± 3^a^
During peak response	10 ± 2^b^	58 ± 4^b^
1 h after end of transportation	79 ± 5^a^	82 ± 5^a^
**Transfer via brailing**	**(F**_**2,6**_ ** = 50.298, p < 0.001)**	(F_2,6_ = 1.730, p = 0.255)
Directly before brailing	88 ± 2^a^	79 ± 5
During peak response	64 ± 4^b^	82 ± 3
1 h after end of brailing	88 ± 4^a^	78 ± 5
**Cage feeding event**	**(F**_**2,6**_ = **31.034, p** = **0.001)**	(F_2,6_ = 0.684, p = 0.540)
Directly before feeding	92 ± 2^a^	80 ± 5
During peak response	65 ± 5^b^	79 ± 3
1 h after end of feeding	85 ± 2^a^	77 ± 6

Statistical analyses were generated using a one-way repeated measures ANOVAs with Bonferroni post hoc test for each stressor and significant differences between GBF and heart rate directly before the initiation of the stressor, during the peak response and 1 h after the end of the stressor are represented by different letters. F- and *p*- values obtained from the statistical analyses are reported in the table and all *p*-values < 0.05 were considered statistically significant. Values of the dependent variables are reported as means ± s.e.m.

Similar to changes observed in response to transportation, GBF initially decreased in response to brailing and the scheduled feeding event (~14% and 27%, respectively), and then recovered to reach levels similar to those observed prior to each practice (Fig. 2A, Table 1). In contrast, heart rate was not significantly affected by brailing or the scheduled feeding event and ranged between ~77 and 82 beats min^−1^ (Fig. 2B, Table 1)

After being subjected to a series of common aquaculture practices on day 0, mean GBF and heart rates (85 ± 3% and 81 ± 4 beats min^−1^, respectively) were significantly higher at the end of the day when compared to levels observed at the beginning of the day (GBF: 53 ± 8%, t_3_ = −3.798, *p* = 0.032; heart rate: 74 ± 3 beats min^−1^, t_3_ = −5.471, *p* = 0.012; Fig. 2).

### Long-term recordings of cardiovascular responses of freely swimming trout in sea cage

**Figure 3:**
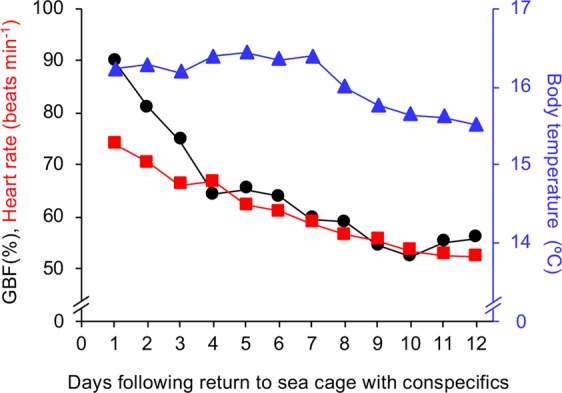
Long-term cardiovascular responses of freely swimming rainbow trout in the sea cage. Mean gastrointestinal blood flow (GBF; black circles and line), heart rate (red squares and line) and body temperature (blue triangles and line) of freely swimming and relatively undisturbed rainbow trout (n = 2). Means were calculated from daily recordings between 07:00 and 14:30.

Daily means of GBF and heart rate of trout (n = 2) from which we managed to obtain reliable long-term recordings of cardiovascular variables in the sea cage are presented in Fig. 3. GBF and heart rate of trout were still substantially elevated (~90% and ~74 beats min^−1^, respectively) the day after being subjected to a series of common aquaculture practices (day 1, Fig. 3). Notably, during the first 7 days in the sea cage, body temperature (and therefore water temperature) remained stable at ~16.3 °C. During these 7 days, mean GBF decreased from ~90% to ~60% (with the majority of the reduction occurring in the first 4 days) and mean heart rate decreased to ~59 beats min^−1^ (Fig. 3). Over the next 5 days (days 8 to 12), mean body temperature decreased by ~0.8 °C, while mean GBF fluctuated between ~52% and ~60% and mean heart rate decreased further to ~52 beats min^−1^ (Fig. 3).

**Figure 4:**
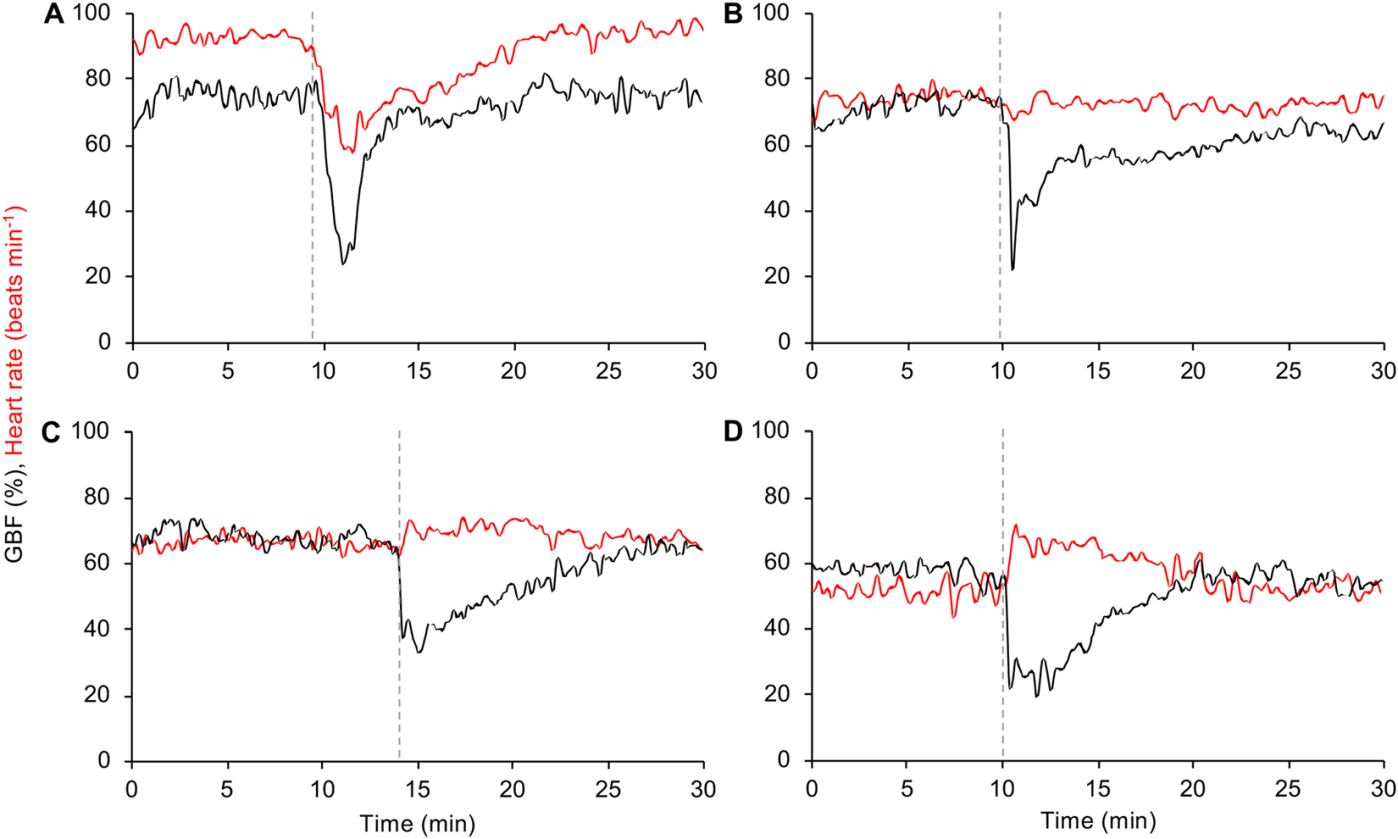
Cardiovascular responses to activity differ in rainbow trout at different stages of recovery. Examples of gastrointestinal blood flow (GBF; black line) and heart rate (red line) responses to activity in a rainbow trout with mean heart rates prior to each burst of activity of (**A**) 93 beats min^−1^, (**B**) 75 beats min^−1^, (**C**) 67 beats min^−1^, and (**D**) 52 beats min^−1^. The beginning of each burst of activity is marked by a grey dashed line.

Inspection of raw traces from both individuals throughout the relatively undisturbed 12-day period revealed that GBF responses during bursts of activity were very consistent (*i*.*e*. relatively rapid initial decrease in GBF followed by a gradual return to levels observed prior to the burst of activity, see examples in Fig. 4A–D). The only known disturbance during this period was the daily approach of the feeding boat and subsequent feeding event (from day 1 to day 10), which consistently reduced GBF by 33 ± 1% and 35 ± 2% in individual 1 and 2, respectively. Close analysis of traces following feeding events did not reveal any postprandial increases in GBF. Despite the fish being left undisturbed for the rest of the time, relatively frequent, and often substantial, activity-induced reductions in GBF were observed in both individuals (individual 1: 84 separate bursts of activity, mean GBF reductions of 25 ± 9%, maximum GBF reduction of 65%; individual 2: 110 separate bursts of activity, mean GBF reductions of 22 ± 6%, maximum GBF reduction of 56%).

**Figure 5:**
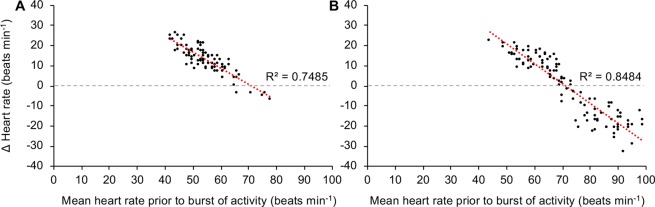
Relationship between mean heart rate and subsequent heart rate response of rainbow trout during activity. Correlation between mean heart rate prior to a burst of activity and the subsequent heart rate response during activity in two rainbow trout with long-term recordings (**A** = individual 1, **B** = individual 2). A Spearman’s rank-order correlation was run to assess the relationship between the two variables for each individual.

In contrast to the consistency of the GBF response, heart rate responses during bursts of activity differed substantially, possibly depending on the individual’s stage of recovery from preceding stressors as indicated by the gradual reduction in mean heart rate over time (*i*.*e*. from day 1 to 7; Fig. 3). When mean heart rates prior to a burst of activity were >70 beats min^−1^, trout displayed a bradycardic response (*i*.*e*. decrease in heart rate, Fig. 4A,B). As mean heart rates prior to a burst of activity decreased, the heart rate response gradually changed from a bradycardic response to a tachycardic response (*i*.*e*. increase in heart rate, Fig. 4C,D). Consistent with these observations of heart rate responses to bursts of activity, there were statistically significant and strong negative correlations between mean heart rate prior to a burst of activity and the subsequent change in heart rate for both individuals (individual 1: *r*_s(82)_ = −0.87, *p* < 0.001, Fig. 5A; individual 2: *r*_s(108)_ = −0.92, *p* < 0.001, Fig. 5B). Indeed, the mean heart rate prior to activity bursts explained 75% and 85% of the variation in heart rate responses to activity bursts for trout 1 and 2, respectively (Fig. 5A,B).

### Cardiovascular responses of freely swimming trout during a series of common aquaculture practices associated with transporting fish from sea cage to slaughterhouse

**Figure 6:**
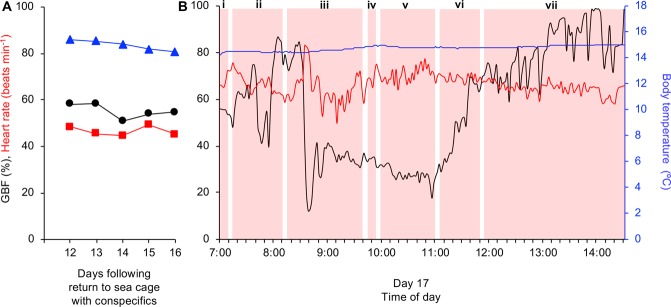
Cardiovascular responses of an individual rainbow trout during the last days in in the sea cage with conspecifics, including a series of common aquaculture practices associated with transporting trout to the slaughterhouse. (**A**) Mean gastrointestinal blood flow (GBF; black line and circles), heart rate (red line and squares) and body temperature (blue line and triangles) of an individual rainbow trout during the last 5 days (days 12–16) in the sea cage with conspecifics (means were calculated from daily recordings between 07:00 and 14:00). (**B**) GBF (black line), heart rate (red line) and body temperature (blue line) of the same individual during a series of common aquaculture practices associated with transporting trout from the sea cage to the slaughterhouse on day 17 (means were calculated every 2 minutes). During this day, (i) the well-boat arrived at the sea cage, (ii) trout were initially crowded at a low density, and (iii) then crowded at a high density. (iv) Trout were then brailed to a well-boat and (v) transported to the slaughterhouse. (vi) Trout remained in a stationary well-boat before (vii) being transferred to a holding pen for slaughter the next day. Red shaded areas represent the duration of each event.

Body temperature of the one trout with good recording signals throughout the entire study gradually decreased from ~15.5 °C to ~14.5 °C during the final days in the sea cage (*i*.*e*. days 12–16; Fig. 6A). During this period, relatively minor fluctuations were observed in the daily means of GBF (between 55 and 58%) and heart rate of this individual (between 45 and 49 beats min^−1^; Fig. 6A).

When comparing mean GBF and heart rate of this individual between 07:00 and 07:15 on days 12–16 (Fig. 6A) with mean GBF and heart rate on day 17 for the same time period, it seems that the arrival of the well-boat at the sea cage at 07:00 most likely caused a slight reduction in GBF from ~58% to ~54% and a substantial elevation in heart rate from ~45 to ~70 beats min^−1^ (see i in Fig. 6B). During low density crowding, a clear acute stress event (*i*.*e*. rapid reduction in GBF, elevation in heart rate) can be observed between 07:40 and 08:00 (see ii in Fig. 6B), which is most likely due to the individual being in close proximity of other trout being captured with a hand net for blood sampling in a separate study^17^. High density crowding initially induced a substantial decrease in GBF from ~85% to ~12% and increase in heart rate to a maximum of ~83 beats min^−1^ (see iii in Fig. 6B). Following the initial response to high density crowding, GBF remained at a reduced level (~35%) and gradually decreased further during successive stressors (*i*.*e*. brailing and transportation) to reach levels below 20% (Fig. see iv-v in 6B). In contrast, following the initial response to high density crowding, heart rate decreased to ~50 beats min^−1^ before gradually increasing during successive stressors (*i*.*e*. brailing and transportation) to reach a level of ~75 beats min^−1^ (see iv-v in Fig. 6B). When held in the stationary well-boat, as well as during recovery in the holding cage by the slaughterhouse, GBF rapidly increased to reach maximum values towards the end of the day (100%), whereas heart rate gradually decreased (see vi-vii in Fig. 6B).

## Discussion

This is the first study to provide continuous and simultaneous recordings of blood flow, heart rate, activity and body temperature in freely swimming fish for extended periods of time. The use of multivariate bio-loggers allowed us to continuously monitor rainbow trout in an aquaculture setting and provided unique insights into the integrative physiological and behavioural principles governing responses of unrestrained fish to anthropogenic and/or environmental stressors.

Continuous monitoring of GBF in freely swimming rainbow trout when subjected to a range of common farming practices (*e*.*g*. crowding, brailing and well-boat transport), as well as during relatively undisturbed periods, clearly demonstrated that acute aquaculture-related stress and/or intense activity consistently resulted in relatively rapid, and often substantial, decreases in GBF. This response in freely swimming trout is consistent with observations from laboratory studies on a range of fish species including rainbow trout^29,31,32^, Chinook salmon (*Oncorhynchus tshawytscha*)^33,34^, sea bass (*Dicentrarchus labrax*)^35^, sturgeon (*Acipenser transmontanus* and *Acipenser medirostris*)^4,25^, Antarctic notothenioid fish (*Pagothenia borchgrevinki*)^36^, and dogfish (*Squalus acanthias*)^37^. Since fish cannot fully perfuse all tissues simultaneously, regional blood flow needs to be tightly regulated and prioritised to match the specific metabolic demands of different tissues with respect to the conditions that the fish currently experiences^27^. Thus, the reduction in GBF during acute stress and/or intense activity is an example of where blood flow to the gastrointestinal tract is not a priority and blood flow is instead allocated to other oxygen demanding organs and tissues such as the heart and swimming musculature^26–28^.

In contrast to most laboratory studies that use traditional “hard wired” methods, the physiological recovery from a combination of stressors could be continuously monitored over an extended period of time in the present study. This provided unique insights into potential differences between the recovery from acute aquaculture-related stress-induced reductions in GBF *vs*. spontaneous activity-induced reductions in GBF of relatively undisturbed fish. While both acute aquaculture-related stress and spontaneous activity resulted in transient reductions in GBF, recovery from stressful handling practices subsequently involved a substantial and prolonged gastrointestinal hyperemia far beyond the level observed prior to the stressor (*i*.*e*. GBF approached a maximum level of 100% and took >4 days to recover). Whereas, following the transient reduction in response to spontaneous activity, GBF gradually returned to levels observed prior to the stressor and no long-term effects were observed. The gastrointestinal hyperemia observed in trout during recovery from acute aquaculture-related stressors is most likely associated with an increased oxygen demand of the already highly metabolically active gastrointestinal tract^29^. Thus, this physiological response may have implications for the aerobic and circulatory capacity of trout to perform other essential processes such as locomotion, digestion, osmoregulation and growth.

The underlying drivers for the prolonged and substantial gastrointestinal hyperemia following acute stress is presently unknown. However, gastrointestinal epithelial damage is well documented in both fish and mammals following acute and chronic stress, and has been demonstrated to impair the ability of intestinal epithelium to maintain selective permeability (*i*.*e*. intestinal barrier function)^38–43^. For example, in both rainbow trout and Atlantic salmon (*Salmo salar*), exposure to acute anthropogenic stressors functionally impaired the intestinal barrier for up to 48 h^31,38,43^. Thus, the prolonged gastrointestinal hyperemia following exposure to anthropogenic stressors may be required to repair stress-induced damage to the gastrointestinal tract by supplying oxygen and nutrients to gastrointestinal tissues whilst removing metabolic waste products. This explanation seems most plausible as other environmental and/or physiological processes that are known to significantly elevate GBF in rainbow trout such as temperature increases^4,29^, elevated salinity^44,45^, and digestive processes^32,45,46^ can be ruled out. This is because water temperature was relatively stable when trout were exhibiting the gastrointestinal hyperemia (~16.3 °C), the salinity of the Djurholms Sound is too low (~5–6 ppt), and the trout were fasted prior to being subjected to stressful handling practices.

Interestingly, in mammals, the gastrointestinal tract is very sensitive to transient reductions in GBF, and even a 20% reduction in flow can result in intestinal ischemia and an impaired intestinal barrier function^47,48^. However, we have previously demonstrated in rainbow trout that even complete occlusion of GBF for 15 min does not impair intestinal barrier function, which suggests that stress-induced damage to the gastrointestinal tract in fish must involve other factors of the physiological stress response^31^. In the present study, when fish were left undisturbed, reductions in GBF associated with spontaneous activity bursts occurred relatively frequently and were often substantial (*i*.*e*. reductions in GBF of up to 65%). However, in contrast to the GBF response following acute stress, no gastrointestinal hyperemias were observed following GBF reductions associated with spontaneous activity in undisturbed fish. If indeed an elevated GBF is required to repair stress-induced intestinal damage, then the lack of this response in undisturbed fish may indicate that the substantial reductions in GBF following voluntary bursts of activity do not result in intestinal damage, which would further support previous findings^31^.

Assuming that the gastrointestinal tract of trout was maximally perfused (*i*.*e*. 100%) when the highest values were recorded during the present study (*i*.*e*. during the peak of the post-stress gastrointestinal hyperemia), then mean GBF of trout ranged between 50% and 65% of maximal capacity following recovery from the series of stressful handling practices. The biological significance of this is that trout had the capacity to increase GBF by at least 35% to 50% following recovery for physiological processes requiring an increase in blood flow. One such physiological process is the post-prandial increase in GBF that occurs during the processing and digestion of a meal^28^. Although trout in the sea cage were fed once daily from day 1 to day 10, no signs of post-prandial increases in GBF were observed during this period. The absence of post-prandial increases in GBF could be due to a number of reasons such as i) the instrumented trout were not feeding, ii) the amount of food ingested was not enough to induce a clear post-prandial increase in GBF, or iii) voluntary feeding fish do not exhibit the substantial post-prandial response as has been documented with gavage fed fish in the laboratory^28^. Unfortunately, with the bio-loggers used in the current study, we are unable to provide a definitive answer to this, but the lack of a post-prandial increase in GBF was most likely due to one of the first two explanations, as individual body masses slightly decreased by ~4% during the course of the study indicating limited to no feed intake in the instrumented fish. Future directions for investigating post-prandial responses of voluntarily feeding fish is to use bio-logging or bio-telemetric devices that continuously record GBF in combination with sensor-based devices measuring parameters such as jaw-motion, swimming depth and motion, or opercular pressure transients, which can provide the necessary information for closely monitoring individual feeding events^7,49,50^.

In parallel with the present study, another study implanted 20 rainbow trout (2082 ± 166 g) with STAR-ODDI heart rate bio-loggers to measure heart rate and body temperature every 10 min throughout the same experimental protocols^17^. Following exposure to the initial series of common farming practices (*i*.*e*. on day 0), it took approximately 4 days for the trout in that study to recover as indicated by a reduction and plateau in resting heart rates (*i*.*e*. mean of the lowest 20% of heart rate values every 24 h) and the emergence of a clear circadian rhythm in heart rate^17^. In the present study, the bio-loggers were programmed to record for only 7.5 h per day to ensure that there was sufficient battery power and memory space for the entire study and thus we were unable to analyse resting heart rates or detect circadian heart rate rhythms. However, when comparing the mean heart rates of trout deemed to have recovered in the abovementioned study with mean heart rates of trout in the present study, it seems that the recovery of the latter was slightly prolonged, as heart rates of trout in the two studies were only in the same range from day 7 onwards (*i*.*e*. <60 beats min^−1^). This finding is not surprising as trout in the present study experienced a more invasive surgical procedure during implantation and the implants are substantially larger (mass in air: 85 g *vs*. 11.8 g). This finding also further suggests that the abovementioned lack of a post-prandial increase in GBF was most likely due to a reduced feed intake, as the propensity for individuals to feed is reduced when recovering from surgery^28–30^.

Continuous, high-resolution monitoring of heart rate before and after recovery in the present study revealed that the heart rate responses of fish to anthropogenic and/or environmental stressors can differ substantially depending on the physiological state of the animal^18,21–23^. In contrast to the consistent GBF response to acute stress and/or intense activity (*i*.*e*. always induced a relatively rapid and substantial reduction in GBF), heart rate responses differed substantially depending on the mean heart rate of trout prior to acute stress and/or intense activity. For example, in response to each of the stressful handling practices on day 0, heart rate of trout did not increase as previously described^17–21^, but instead decreased or remained unchanged. When closely examining heart rate responses of trout throughout the study, a statistically significant and strong negative correlation existed between mean heart rate prior to acute stress and/or intense of activity and the subsequent change in heart rate. In fact, the mean heart rate prior to intense activity explained 75% and 85% of the variation in heart rate responses to activity in the two individuals examined. When the mean heart rate was approximately higher than 70 beats min^−1^ (*e*.*g*. during day 0), trout generally displayed a bradycardic response to acute stress and/or intense of activity, whereas when the mean heart rate decreased, the heart rate response to the same stressors changed to a tachycardic response. Interestingly, when the mean heart rate of trout in the present study was similar to those of trout deemed to have recovered in Brijs *et al*.^17^, spontaneous bouts of intense activity induced clear tachycardic responses (see example in Fig. 4D). In contrast to the bradycardic or lack of heart rate responses to stressful handling practises observed on day 0, the fully recovered individual trout exhibited clear tachycardic responses to crowding and transportation on day 17, which is consistent with previous findings^17^. Currently, the underlying mechanisms and biological/ecological significance of the apparent negative heart rate scope of individuals with elevated mean heart rates remains unknown and warrants further investigation.

## Conclusions

The present study demonstrates that the application of bio-loggers provides a powerful tool for the further development of management protocols aiming to reduce stress of farmed fish and the subsequent ethical and economic consequences for the aquaculture industry. The use of these devices in freely swimming fish allow the continuous collection of data over time in the same individual as opposed to the ‘snapshot’ provided by more traditional measures (*e*.*g*. stress hormones, blood and tissue samples, *etc*). Thus, by using these devices, potentially fewer experimental animals are necessary (thus keeping in line with the guiding principles of the 3Rs with regards to Reduction). This is because researchers are able to avoid the large individual variation associated with repeated sampling on different individuals, as well as the accumulative risk of sampling errors with repeated sampling. Although the implantation of these multivariate bio-loggers is currently technically challenging (*i*.*e*. requires specialised surgical training and extensive knowledge of the anatomy/physiology of fish), future technological development of these devices may result in sensor and device designs that allow for a more user-friendly and potentially less invasive implantation procedure.

The present study provides unique insights on physiological and behavioural responses of freely swimming fish, which have previously been impossible to obtain with ‘hardwired’ individuals confined in a laboratory setting. The consistency of the GBF response to acute stress and activity (*i*.*e*. relatively rapid and substantial decreases in GBF), as well as the respective differences in recovery following the reduction in GBF (*i*.*e*. rapid recovery following activity *vs*. a substantial and prolonged gastrointestinal hyperemia following an aquaculture-related stress event), demonstrates that continuous monitoring of GBF could be a useful indicator for stress to assess the welfare of farmed fish. Long term recordings of heart rate clearly demonstrated that heart rate responses to acute stress or intense activity differ depending on the physiological state of the animal (*i*.*e*. chronically stressed fish tend to exhibit bradycardic responses to acute stress or activity whereas ‘unstressed’ fish tend to exhibit tachycardic responses). This means that researchers utilising heart rate as an indicator of stress for assessing the welfare of farmed fish need to be aware of the physiological and behavioural constraints that affect heart rate and thoroughly evaluate the range of heart rates normally exhibited by their model species. Individuals that deviate from this pattern should be critically examined, as the constraints related to elevated resting heart rates (most likely due to negative stress) may bias the results and lead to erroneous interpretations and conclusions.

Future research regarding fundamental stress biology in fish, as well as the improvement of fish welfare in aquaculture, should aim to shift from laboratory studies to field studies in order to get a more comprehensive understanding of the environmental factors and integrative physiological and behavioural principles that govern responses to anthropogenic and/or environmental stressors of unrestrained fish. Although the technology is not yet available, further research should be aimed at developing these devices so that they are able to transmit the high-resolution physiological and behavioural data in ‘real-time’ (*e*.*g*. acoustic-based telemetry). From an aquaculture perspective, this would prevent the need to physically retrieve the data (an increasingly difficult task in modern fish farms that can have >200,000 fish per sea cage), while also allowing commercial fish farmers to take action directly based on the ‘on-line’ information available from focal animals.

## Methods

### Location, experimental animals and ethical statement

The study was conducted at the Brändö Lax AB facilities (Brändö, Åland Islands, Finland) between the 31^st^ of August and 27^th^ of September, 2016. Only female rainbow trout were used in this study, as only this sex is farmed at Brändö Lax AB. Trout were initially kept with ~5000 conspecifics (total biomass: ~9500 kg) in a circular sea cage (diameter: 40 m, depth: 4 m) anchored in the Djurholms Sound. Animal care and all physiological experimental procedures were performed in accordance with guidelines and regulations that were approved by the Åland Provincial government project approval committee (decision 2/2016).

### Programming, data retrieval and analysis of data from multivariate bio-loggers

The fully implantable multivariate bio-logger system used in this study included an implant, battery pack, data logger and measuring electrodes (Transonic EndoGear3 Implant and custom-built Logger, EG3-VXSXT-M23 and EG3-DTl, respectively, Transonic Systems Inc, Ithaca, New York, USA). For simplicity’s sake, the entire system is hereafter referred to as a ‘bio-logger’ (see Fig. 1A,B). The bio-logger was originally developed and tested in pigs and alligators^3^, and later validated for use in fish^4,24^. Specific details and validation tests for the multivariate bio-telemetric components of the system can be found at https://www.transonic.com/product/endogear3/, as well as in the abovementioned studies^3,4,24^. The bio-loggers were programmed with the EndoGear3 Base Station and the accompanying software (EG108 and EndoGear EGUI 2.20, Transonic Systems Inc., Ithaca, New York, USA) to record and store GBF, ECG, acceleration and body temperature at a daily sampling frequency of 200 Hz between 07:00 and 14:30. Only 7.5 h per day were recorded to ensure that there was sufficient battery power and memory space for the total duration of the study (18 days), whilst the specific times of day were chosen according to the daily operational schedule of the aquaculture facility (*i*.*e*. time of day when trout would be subjected to a range of common aquaculture practices).

Blood flow (*i*.*e*. GBF) was measured using a 2.5 mm Doppler flow probe (20 MHz). ECG was obtained via a bio-amplifier that uses two measuring electrodes. Acceleration and body temperature were obtained via a 3D-accelerometer and digital temperature sensors, respectively, which were located within the bio-telemetric component of the bio-logger. The bio-logger was calibrated using the EndoGear3 Base Station user interface and 3-step calibrations were employed for both the blood flow channel and the temperature channel. The bio-logger contains a real-time clock with a drift of ±1 min month^−1^. All times are reported in Eastern European Summer Time (EEST or UTC + 2). The total mass in air of the bio-logger (*i*.*e*. implant, battery pack, data logger and measuring electrodes) was ~85 g (*i*.*e*. ~3% of the body mass of all individuals) and fitted easily within their abdominal cavity.

The recorded data were downloaded to a PC computer and converted to ASCII code using the software EndoGear BtoASCII 3.10 (Transonic Systems Inc Ithaca, New York, USA) and then imported into LabChart Pro software (v7.3, ADInstruments, Castle Hill, Australia). Heart rates were determined from ECG using the ECG module in LabChart. Raw GBF signals were converted into relative values to allow comparisons between individuals. This was achieved by dividing mean raw GBF values (calculated every 2 min throughout the entire GBF recordings for each individual) with the highest mean raw GBF value calculated for that individual during the study and then multiplying by 100 (*e*.*g*. 100% GBF = highest mean value calculated for a specific individual throughout its recordings). The acceleration data was only used to identify burst of activity^6^.

### Surgical implantation of the multivariate bio-loggers

On the 31^st^ of August, trout were lightly crowded in the sea cage and ~40 individuals were brailed into a smaller rectangular holding cage (width: 2 m, length: 6 m, depth: 1 m), which was subsequently towed by boat to the Brändö Lax AB facilities by boat. Trout were kept and fasted in this location for 1 week. On the 6^th^ of September, 4 trout were randomly selected for the surgical implantation of the bio-loggers (individuals 1, 2, 3 and 4 had body masses of 2688 g, 3424 g, 2770 g and 2880 g, respectively, mean body mass: 2941 ± 166 g). To surgically implant the bio-loggers, trout were individually anaesthetized in water from the Djurholms Sound (5–6 ppt) containing 150 mg L^−1^ ethyl-3-aminobenzoate methanesulphonic acid (MS222, Sigma-Aldrich Inc., St. Louis, Missouri, USA) and buffered with 300 mg L^−1^ NaHCO_3_. When ventilation ceased and individuals were deemed to have reached surgical anaesthesia, they were placed on their left side on an operating table covered with soft, water-soaked foam. To maintain anaesthesia and a sufficient oxygen supply, their gills were continuously irrigated with aerated seawater containing 75 mg L^−1^ MS222 and buffered with 150 mg L^−1^ NaHCO_3_ at 10 °C.

The first step in the surgical procedure was to make a ~40 mm incision ventrodorsally starting from the base of the pectoral fin on the left side of the fish. The bio-logger was then inserted and carefully positioned within the abdominal cavity (Fig. 1A). GBF was measured from the coeliacomesenteric artery, which is the first caudal branch of the dorsal aorta that divides progressively to supply the stomach, intestine, liver and gonads^27^. The artery was accessed via the incision and dissected free using blunt dissection, ensuring that surrounding blood vessels and nerves remained intact. The Doppler flow probe was then placed around the vessel, approximately 10 mm proximal to the bifurcation from the dorsal aorta (Fig. 1B), and closed using the integrated silk sutures. To record the ECG, the two external measuring electrodes were tunnelled out ventrally from within the abdominal cavity using a 23-gauge needle. The electrode tips were then inserted beneath the skin on the ventral surface between the pectoral fins in a medial position, with one electrode directed posteriorly and the other directed anteriorly with the tip close to the heart (Fig. 1B). The electrodes were secured to the skin using 3–0 silk sutures (Ethicon Inc., Somerville, New Jersey, USA). After ensuring that the bio- logger components were well positioned, and all the abdominal organs had been restored to their places of origin, the incision was closed with interrupted stitches using 3–0 sterile monofilament non-absorbable Prolene suture material (Ethicon Inc, Somerville, New Jersey, USA). A mixture of Orabase® (a protective paste, ConvaTec, Bromma, Sweden), Pevaryl® (an antifungal agent, McNeil Sweden AB, Solna, Sweden) and Bacibact® (an antibacterial agent, Orion Corporation, Espoo, Finland) was applied on the surface of the wound. A dose of Baytril® vet (10 mg kg^−1^, Bayer Healthcare, Berlin, Germany), a broad-spectrum antibiotic, was injected intraperitoneally. Following surgery, which took between 60–90 min, trout were returned to the smaller rectangular holding cage to recover for 2 days. Upon return to the holding cage following surgery, all trout emerged relatively quickly from surgical anaesthesia and were observed to swim around the enclosure within 20 minutes. Inspection of trout at the end of the study revealed that all of the wounds had remained closed. Unfortunately, signals from some bio-loggers were lost during the study and this is reflected in the declining number of individuals used for each subsequent analysis described below.

### Experimental protocols

#### Cardiovascular responses of freely swimming trout during a series of common aquaculture practices associated with transporting fish from slaughterhouse to the sea cage

On the 9^th^ of September (3 days after surgical instrumentation), trout implanted with the bio-loggers, as well as the remaining uninstrumented trout, were returned to the sea cage containing ~5000 conspecifics. During this day (referred to as day 0), trout were subjected to a series of common aquaculture practices such as transportation (holding cage containing trout was towed slowly by a boat from 07:30 to 08:30), transfer from holding cage to sea cage via brailing (from 10:10 to 10:40), and a scheduled feeding event (a feeding boat propels pellets into the sea cage resulting in feeding frenzy of cage inhabitants from 13:00 to 13:30). Mean GBF, heart rate, body temperature, and acceleration/motion of trout were analysed every 2 minutes between 07:00 and 14:30 (n = 4 with good signals).

#### Long-term recordings of cardiovascular responses of freely swimming trout in sea cage

Between the 10^th^ and 25^th^ of September (4 and 19 days after surgical instrumentation), cardiovascular variables, activity and body temperature of trout were recorded in the sea cage. During this 16-daay period, daily means for GBF, heart rate and body temperature of trout were calculated from the recordings (n = 2 with good signals for days 1–12; n = 1 with good signals for days 13–16). In addition, during bursts of activity that resulted in a ‘clear’ reduction in GBF (*i*.*e*. >15%), the respective heart rate and GBF responses were analysed for each individual throughout their entire recordings. This was carried out by calculating the mean of each parameter directly prior to the burst of activity, as well as the change in each parameter in response to the burst of activity.

Trout within the sea cage were fed *ad libitum* once per day with commercial trout pellets (BioMar, Aarhus, Denmark) according to the normal feeding regime at the farm from day 0 to 10. In accordance with pre-slaughter procedures employed at Brändö Lax AB, the trout were fasted from day 11 onwards (*i*.*e*. 1 week before transport to the slaughterhouse).

#### Cardiovascular responses of freely swimming trout during a series of common aquaculture practices associated with transporting fish from sea cage to slaughterhouse

On the 26^th^ of September (day 17), the well-boat arrived at the sea cage at 07:00. At 07:15, the trout were crowded at a low density to allow the capture of trout with a hand net for blood sampling in a separate study^17^. At 08:15, the bottom of the sea cage was elevated to further increase the level of crowding. At 09:45, trout were transferred from the sea cage to the well-boat using a brail net. Between 10:00 and 11:00 am, trout were transported from the sea cage to the Brändö Lax AB slaughter facilities. Trout remained in a stationary well-boat at the slaughter facilities between 11:05 and 11:50, before being transferred to a holding pen via a water chute at 11:55, where after they were left undisturbed until slaughter the next day. Mean GBF, heart rate, body temperature, and acceleration/motion of trout were analysed every 2 minutes between 07:00 and 14:30 (n = 1 with good signals).

### Statistical analyses

Statistical analyses were performed with SPSS Statistics 22 (IBM Corp., Armonk, NY, USA). One-way repeated measures ANOVAs with Bonferroni post hoc tests were used to statistically analyse changes in GBF and heart rate of trout in response to common aquaculture practices (*i*.*e*. transportation, brailing and a feeding event). Mean GBF and heart rate were calculated from 2 min periods directly before the initiation of each event, during the peak response (defined as the 2 min period where GBF was at its lowest during each event) and 1 h after the end of each event. Data used in this statistical analysis were assessed for normality, outliers and sphericity.

A paired-samples t-test was used to statistically analyse differences in GBF and heart rate of trout at the beginning and end of day 0. This was carried out to statistically analyse the cumulative effects of multiple aquaculture practices performed in one day on GBF and heart rate of trout. Mean GBF and heart rate were calculated from a 30 min period at the beginning (*i*.*e*. 07:00 to 07:30) and end of the day (*i*.*e*. 14:00 to 14:30). Data used in this statistical analysis were assessed for normality and outliers.

A Spearman’s rank-order correlation was run to assess the relationship between mean heart rates prior to a burst of activity and the subsequent heart rate response of two individuals with long-term recordings. Data used in this statistical analysis were assessed to ensure that there was a monotonic relationship between the two variables.

F-, t- and *p*- values obtained from the statistical analyses are reported throughout the text and all *p*-values < 0.05 were considered statistically significant. Values for the dependent variables are reported as means ± s.e.m. unless otherwise stated.

### Ethics statement

Animal care and all physiological experimental procedures were performed in accordance with guidelines and regulations that were approved by the Åland Provincial government project approval committee (decision 2/2016).

## Supplementary information

Dataset 1

## Data Availability

The datasets supporting this article have been uploaded as part of the Supplementary Material.
