# Supplementary material for: Remote physiological monitoring provides unique insights on the cardiovascular performance and stress responses of freely swimming rainbow trout in aquaculture
Source: Sci Rep. 2019 Jun 24;9:9090. doi: 10.1038/s41598-019-45657-3 (PMC6591390; doi:10.1038/s41598-019-45657-3)
Supplement: Supplementary file 1 — Dataset 1 [file 41598_2019_45657_MOESM1_ESM.pdf]

Remote physiological monitoring provides unique insights on the cardiovascular performance and stress responses of freely swimming rainbow trout in aquaculture

SUPPORTING DATA FOR MANUSCRIPT

Jeroen Brijs[1], Erik Sandblom[2], Michael Axelsson[2], Kristina Sundell[2], Henrik Sundh[2], Anders Kiessling[3], Charlotte Berg[1]and Albin Gräns[1]*

[1] Department of Animal Environment and Health, Swedish University of Agricultural Sciences, Skara, SE-532 31, Sweden
[2]Department of Biological and Environmental Sciences, University of Gothenburg, Gothenburg, SE-405-30, Sweden
[3]Department of Animal Nutrition and Management, Swedish University of Agricultural Sciences, Uppsala, SE-750 07, Sweden

Cardiovascular responses of rainbow trout during a series of common aquaculture practices following the surgical implantation of bio-loggers (Fig. 2 and Table 1)

RELATIVE GASTROINTESTINAL BLOOD FLOW (%)

Events	Individual 1	Individual 2	Individual 3	Individual 4
Before transportation	81.32436627	66.47082658	40.893961	62.22062
Transportation initiated	9.984480083	5.875202593	13.1716595	12.6892574
End of transportation	87.32540093	73.90599676	85.4493581	68.2768565
Before transfer	89.39472323	90.29578606	89.3961008	83.741889
Transfer initiated	57.4754268	73.27795786	68.8540181	56.0562365
End of transfer	85.30781169	94.36790924	95.2924394	77.4333093
Before feeding	92.55043973	93.39546191	85.3542558	96.0346071
Directly after feeding	51.83652354	75.34440843	59.8668569	72.2782985
End of day	79.4619762	89.10048622	81.4550642	88.0677722

HEART RATE (BEATS MIN^{-1})

Events	Individual 1	Individual 2	Individual 3	Individual 4
Before transportation	64.4377	70.4118	67.3917	78.0745
Transportation initiated	50.2701	57.8675	55.3175	69.7521
End of transportation	70.0227	94.4182	78.4135	84.9477
Before transfer	66.5993	91.0444	77.3602	80.9862
Transfer initiated	74.2085	89.7031	80.6059	82.9243
End of transfer	64.952	85.0783	79.4136	83.7638
Before feeding	67.4404	90.0645	78.2845	84.0226
Directly after feeding	72.4241	85.3813	78.1508	79.1498
End of day	59.9359	86.0962	78.9278	82.5036

Long-term cardiovascular responses of freely swimming rainbow trout in the sea cage (Fig. 3)

INDIVIDUAL 1

Day	Relative GBF (%)	Heart Rate (beats min^{-1})	Temperature (°C)
1	97.61683049	62.76159333	16.148
2	84.54906018	59.41358	16.21466667
3	77.63062597	54.95409333	16.14
4	66.22176237	58.36408667	16.35866667
5	64.80082773	51.24646	16.416
6	67.05294016	50.51934667	16.30133333
7	54.5645801	48.35616667	16.37266667
8	58.82393516	46.99187333	15.998
9	53.52647008	45.97104	15.77333333
10	50.57768581	46.2217	15.64266667
11	58.15830316	46.06086	15.624
12	58.31350233	48.4722	15.46733333

INDIVIDUAL 2

Day	Relative GBF (%)	Heart Rate (beats min^{-1})	Temperature (°C)
1	82.26634252	85.27316667	16.308
2	77.61885467	81.51934	16.33666667
3	71.98541329	77.46222667	16.24333333
4	62.45137763	75.30125333	16.41533333
5	65.77120475	73.2249	16.46333333
6	60.71447866	71.78089333	16.38933333
7	64.69611021	69.49721333	16.4
8	59.43679092	66.09208	15.984
9	55.60237709	65.11649333	15.768
10	54.26796326	60.68089333	15.656
11	52.21907077	59.35549333	15.59466667
12	53.54132901	56.28865333	15.562

Relationship between mean heart rate and subsequent heart rate response of rainbow trout during activity (Fig. 5)

INDIVIDUAL 1		INDIVIDUAL 2	
Mean heart rate prior to activity (beats min^{-1})	Change in heart rate in response to activity (beats min^{-1})	Mean heart rate prior to activity (beats min^{-1})	Change in heart rate in response to activity (beats min-1)
78	-7	76	-21
75	-5	79	-18
71	-4	78	-15
65	0	81	-23
66	-4	83	-17
76	-4	82	-17
67	5	83	-18
65	4	99	-17
66	7	95	-20
63	10	95	-29
66	4	82	-22
61	4	86	-21
58	7	79	-14
62	8	82	-15
62	9	83	-18
63	13	90	-22
61	7	98	-13
59	15	99	-20
59	7	86	-21
61	9	94	-20
60	13	88	-17
57	9	75	-18
53	17	87	-11
61	10	89	-21
56	15	89	-26
58	10	89	-24
56	14	90	-20
59	11	90	-16
55	16	91	-14
58	9	92	-33
54	15	94	-18
54	16	90	-21
57	15	91	-16
58	13	94	-21
55	8	88	-9
52	12	90	-13
53	13	87	-11
53	20	87	-9
53	21	82	-12
54	11	78	-10
55	9	82	-7
57	15	84	-14
53	12	80	-7
51	8	77	-4
52	10	77	-9
53	12	74	-7
49	15	69	2
54	12	73	2
52	22	70	3
57	8	74	-3
52	21	70	-5
54	21	70	3
54	17	69	0
50	17	70	4
52	18	71	3
49	16	73	0
54	13	69	10
51	15	70	1
46	23	68	10
49	17	72	-2
45	20	67	3
46	20	62	14
50	11	66	7
49	17	68	13
48	15	59	13
45	18	63	11
44	26	65	9
47	25	67	15
49	16	70	-8
49	16	65	13
45	25	64	16
48	10	64	15
42	23	63	9
57	11	68	9
50	14	66	14
48	20	68	7
50	18	66	4
43	23	62	18
47	16	66	5
42	25	63	12
44	17	56	17

48	18	65	8
50	13	62	14
56	11	67	7
		61	17

Relationship between mean heart rate and subsequent heart rate response of rainbow trout during activity (Fig. 5)… CONTINUED

INDIVIDUAL 2 (continued)

Mean heart rate prior to activity (beats min^{-1})	Change in heart rate in response to activity (beats min^{-1})
59	9
58	17
54	18
58	12
58	14
58	19
56	11
60	10
58	8
54	19
54	13
52	15
55	13
56	10
53	21
52	16
55	13
53	15
49	19
49	21
51	16
52	15
54	9
44	22

Cardiovascular responses of an individual rainbow trout during the last days in in the sea cage with conspecifics, including a series of common aquaculture practices associated with transporting trout to the slaughterhouse (Fig. 6)

INDIVIDUAL 1

Day	Relative GBF (%)	Heart rate (beats min^{-1})	Temperature (°C)
12	58.3135023	48.4722	15.4673333
13	58.482497	45.3549333	15.3466667
14	50.9122262	44.7103533	15.1206667
15	53.9989653	49.3235733	14.7486667
16	54.6266598	45.2349733	14.5306667

INDIVIDUAL 1

Day	Relative GBF (%)	Heart rate (beats min^{-1})	Temperature (°C)
0.29166667	55.9234351	66.1604	14.22
0.29305556	55.6647698	65.662	14.43
0.29444444	55.5613037	65.1636	14.45
0.29583333	55.4061045	67.7833	14.49
0.29722222	53.5437144	72.2943	14.5
0.29861111	53.4919814	72.1326	14.5
0.3	52.0951888	72.6375	14.5
0.30138889	48.0600103	75.6298	14.5
0.30277778	55.7682359	73.4244	14.52
0.30416667	61.0967408	72.2399	14.5
0.30555556	63.3212623	70.999	14.5
0.30694444	62.2348681	71.3806	14.5
0.30833333	62.2348681	69.9109	14.5
0.30972222	60.5794102	68.1467	14.5
0.31111111	61.7692706	66.7158	14.5
0.3125	66.0631143	65.1527	14.5
0.31388889	60.2690119	66.1373	14.5
0.31527778	67.5116399	62.167	14.5
0.31666667	74.5990688	62.4325	14.5
0.31805556	74.6508019	64.635	14.5
0.31944444	68.6497672	65.2478	14.5
0.32083333	47.6978789	66.9677	14.49
0.32222222	45.00776	67.3051	14.46
0.32361111	41.1277807	68.8131	14.45
0.325	44.3869633	67.6484	14.45
0.32638889	51.0087946	65.1282	14.44
0.32777778	54.4749095	67.0727	14.45
0.32916667	39.5757889	69.5473	14.44
0.33055556	57.2167615	66.4209	14.44
0.33194444	71.2881531	62.3489	14.44
0.33333333	77.2891878	63.6846	14.46
0.33472222	83.3936886	62.6605	14.48
0.33611111	86.7046042	62.7247	14.45
0.3375	85.61821	62.7737	14.47
0.33888889	84.3766167	62.085	14.45
0.34027778	74.9094671	61.509	14.45
0.34166667	79.1515779	58.6958	14.47
0.34305556	75.685463	61.7155	14.48
0.34444444	72.3228143	62.0593	14.45
0.34583333	74.2886705	59.4086	14.46
0.34722222	77.0822556	59.9423	14.45
0.34861111	78.9446456	61.7105	14.44
0.35	83.2384894	66.1788	14.45
0.35138889	82.7728919	68.3865	14.44
0.35277778	84.5835489	67.0659	14.42
0.35416667	79.6689084	68.7614	14.45
0.35555556	72.8401449	68.0149	14.44
0.35694444	42.6797724	83.0696	14.44
0.35833333	19.7620279	82.5533	14.44
0.35972222	12.2090016	81.2478	14.45
0.36111111	12.7780652	75.6401	14.49
0.3625	16.9167098	63.5619	14.5
0.36388889	28.2462494	63.7147	14.5
0.36527778	36.9891361	66.9229	14.5
0.36666667	41.0760476	70.0547	14.5
0.36805556	27.884118	68.4879	14.53
0.36944444	26.4355923	59.2585	14.56
0.37083333	30.4707708	52.7108	14.6
0.37222222	39.1619245	60.4606	14.62
0.37361111	38.9549922	62.5533	14.62
0.375	41.2829798	62.5102	14.62
0.37638889	39.9896534	60.2473	14.62
0.37777778	39.7309881	58.6496	14.62

0.37916667	37.7133989	62.7212	14.62
0.38055556	35.5406105	61.1472	14.62
0.38194444	33.1608898	49.7331	14.62

Cardiovascular responses of an individual rainbow trout during the last days in in the sea cage with conspecifics, including a series of common aquaculture practices associated with transporting trout to the slaughterhouse (Fig. 6)…CONTINUED

INDIVIDUAL 1 (CONTINUED)

Day	Relative GBF (%)	Heart rate (beats min^{-1})	Temperature (°C)
0.38472222	33.9886187	57.4889	14.63
0.38611111	35.6440766	61.7077	14.64
0.3875	35.0232799	54.8854	14.67
0.38888889	37.1443352	57.7693	14.69
0.39027778	37.6616658	63.9614	14.7
0.39166667	34.5576824	58.4483	14.69
0.39305556	33.2643559	64.0547	14.69
0.39444444	34.4542162	65.1271	14.71
0.39583333	32.6952923	66.4849	14.75
0.39722222	32.1262287	71.2313	14.75
0.39861111	31.7123642	69.9046	14.78
0.4	30.7811692	71.8985	14.81
0.40138889	33.5747543	63.9482	14.81
0.40277778	36.937403	58.251	14.83
0.40416667	34.7128815	64.2183	14.87
0.40555556	33.316089	68.3801	14.88
0.40694444	34.5059493	69.1914	14.88
0.40833333	34.5576824	67.9747	14.88
0.40972222	35.2302121	65.1044	14.91
0.41111111	35.0750129	71.3925	14.94
0.4125	31.0915675	75.0527	14.94
0.41388889	30.8329022	72.5465	14.85
0.41527778	31.8675634	70.0403	14.94
0.41666667	31.8675634	67.5341	14.94
0.41805556	31.0915675	68.3621	14.94
0.41944444	31.8675634	69.4011	14.94
0.42083333	32.6435592	66.5609	14.91
0.42222222	32.436627	68.5722	14.88
0.42361111	30.5742369	68.8599	14.88
0.425	27.7806518	72.675	14.88
0.42638889	26.9529229	65.9371	14.88
0.42777778	25.6078634	72.8137	14.87
0.42916667	27.7806518	64.0893	14.86
0.43055556	28.815313	68.5864	14.82
0.43194444	26.9529229	70.4441	14.81
0.43333333	25.7113295	74.4303	14.81
0.43472222	27.4702535	71.0784	14.81
0.43611111	25.9699948	66.3279	14.81
0.4375	28.3497155	69.0523	14.81
0.43888889	26.9011899	67.0255	14.81
0.44027778	27.004656	71.285	14.81
0.44166667	27.3667874	68.3604	14.81
0.44305556	27.1598551	74.7356	14.81
0.44444444	29.0739783	70.8267	14.81
0.44583333	24.36627	74.0057	14.81
0.44722222	28.2979824	76.1504	14.81
0.44861111	28.2979824	72.1708	14.81
0.45	26.4873254	77.3729	14.81
0.45138889	28.0910502	73.1305	14.81
0.45277778	27.1598551	74.9554	14.79
0.45416667	23.0729436	70.4887	14.81
0.45555556	17.6409726	71.9946	14.81
0.45694444	23.9006725	70.9917	14.82
0.45833333	26.7977237	66.959	14.84
0.45972222	28.9705122	69.8623	14.83
0.46111111	32.2296948	71.0392	14.83
0.4625	28.9705122	70.3097	14.84
0.46388889	31.4019659	69.8589	14.83
0.46527778	34.5576824	69.3804	14.81
0.46666667	33.0574237	67.4586	14.81
0.46805556	35.0232799	67.8023	14.81
0.46944444	37.0408691	71.3784	14.81
0.47083333	36.8856699	68.3497	14.81
0.47222222	41.6968443	67.4828	14.81
0.47361111	50.3362649	67.8791	14.81
0.475	49.9224004	70.6126	14.71
0.47638889	45.938955	69.9712	14.81
0.47777778	49.7672012	69.3298	14.81
0.47916667	51.9917227	68.6884	14.81
0.48055556	52.1469219	66.1897	14.81
0.48194444	48.5773409	67.083	14.81
0.48333333	60.1138127	70.9726	14.81
0.48472222	68.8049664	69.2164	14.8
0.48611111	69.5809622	71.3999	14.77

0.4875	67.4081738	68.1262	14.76
0.48888889	66.4769788	70.8823	14.75
0.49027778	66.5804449	67.4697	14.75

Cardiovascular responses of an individual rainbow trout during the last days in in the sea cage with conspecifics, including a series of common aquaculture practices associated with transporting trout to the slaughterhouse (Fig. 6)…CONTINUED

INDIVIDUAL 1 (CONTINUED)

Day	Relative GBF (%)	Heart rate (beats min^{-1})	Temperature (°C)
0.49583333	70.3569581	67.7864	14.75
0.49722222	72.5814796	67.4958	14.75
0.49861111	75.5819969	68.3983	14.75
0.5	74.5473357	69.0437	14.76
0.50138889	70.4604242	67.188	14.77
0.50277778	66.7356441	68.6666	14.77
0.50416667	70.7708226	68.8347	14.77
0.50555556	74.6508019	66.9893	14.77
0.50694444	62.7004656	66.1068	14.79
0.50833333	69.5292292	64.3386	14.81
0.50972222	66.683911	66.4382	14.81
0.51111111	67.2012416	66.8205	14.81
0.5125	67.8220383	64.0171	14.81
0.51388889	71.0812209	65.325	14.81
0.51527778	58.9239524	63.2048	14.81
0.51666667	72.5297465	65.1336	14.81
0.51805556	75.5819969	65.0091	14.81
0.51944444	76.1510605	63.5559	14.81
0.52083333	78.8411795	67.1579	14.82
0.52222222	81.996896	65.4587	14.84
0.52361111	64.6145887	64.1757	14.84
0.525	70.4604242	64.1596	14.83
0.52638889	75.5819969	65.3071	14.85
0.52777778	78.0651837	64.9773	14.87
0.52916667	64.873254	60.2634	14.87
0.53055556	68.7532333	62.2708	14.87
0.53194444	76.6683911	64.8026	14.88
0.53333333	77.444387	63.453	14.88
0.53472222	80.4966374	64.7938	14.88
0.53611111	81.5312985	64.4595	14.75
0.5375	82.1003621	63.2645	14.88
0.53888889	62.3900673	59.1494	14.88
0.54027778	68.7015003	66.1075	14.89
0.54166667	77.8065184	66.0238	14.88
0.54305556	80.7553026	65.9401	14.88
0.54444444	81.6347646	65.8564	14.91
0.54583333	89.9120538	69.524	14.92
0.54722222	92.757372	67.1803	14.93
0.54861111	90.1707191	65.9831	14.94
0.55	87.8944646	64.553	14.94
0.55138889	89.4464563	65.6088	14.94
0.55277778	90.118986	66.1129	14.94
0.55416667	93.688567	64.6487	14.94
0.55555556	94.4645629	67.5922	14.94
0.55694444	94.9301604	62.7159	14.94
0.55833333	94.568029	65.1429	14.94
0.55972222	80.7553026	63.414	14.94
0.56111111	91.7227108	62.4254	14.94
0.5625	87.3254009	66.0894	14.94
0.56388889	88.5669943	66.8677	14.94
0.56527778	76.2545266	62.9634	14.94
0.56666667	85.4630109	63.8275	14.94
0.56805556	90.7915158	65.2542	14.94
0.56944444	89.0843249	66.1436	14.94
0.57083333	88.6187274	61.3541	14.95
0.57222222	93.5333678	64.7006	14.95
0.57361111	96.7408174	67.158	14.96
0.575	96.7925504	63.8838	14.97
0.57638889	76.7201242	63.3258	14.96
0.57777778	88.6187274	65.2787	14.97
0.57916667	98.5514744	64.9923	14.98
0.58055556	97.8789446	65.4428	14.99
0.58194444	98.8618727	65.0633	15
0.58333333	98.6549405	64.9088	15
0.58472222	98.6549405	64.9182	15
0.58611111	96.5338852	61.1018	15
0.5875	82.8246249	58.32	15
0.58888889	73.1505432	58.5099	15
0.59027778	78.9963787	60.1604	15
0.59166667	82.1520952	60.3764	15
0.59305556	91.5675116	61.7582	15
0.59444444	77.3926539	59.2058	15
0.59583333	71.5468184	58.4941	15
0.59722222	82.3072944	58.1119	15
0.59861111	82.6694258	62.8288	15
0.6	83.0315572	64.9816	15
0.60138889	83.3936886	65.2637	15

0.60277778	83.75582	65.5458	15
0.60416667	98.5514744	65.8279	15
